# Clinical Use of a 180-Day Implantable Glucose Monitoring System in Dogs with Diabetes Mellitus: A Case Series

**DOI:** 10.3390/ani12070860

**Published:** 2022-03-29

**Authors:** Antonio Maria Tardo, Concetta Irace, Francesca Del Baldo, Armando Foglia, Federico Fracassi

**Affiliations:** 1 Department of Veterinary Medical Sciences, University of Bologna, Ozzano dell’Emilia, 40064 Bologna, Italy; francesca.delbaldo2@unibo.it (F.D.B.); armando.foglia2@unibo.it (A.F.); federico.fracassi@unibo.it (F.F.); 2 Department of Health Science, University Magna Græcia, 88100 Catanzaro, Italy; irace@unicz.it

**Keywords:** Canine diabetes mellitus, Eversense XL, implantable sensor, long-term continuous glucose monitoring system, flash glucose monitoring system

## Abstract

**Simple Summary:**

A novel continuous glucose monitoring system (CGMS) equipped with a long-term sensor has recently been developed for humans with diabetes mellitus. The sensor is inserted under the skin and continuously measures the glucose in the interstitial fluid over a period of up to 180 days. The aim of this study was to describe, for the first time, the clinical use of this novel CGMS in three diabetic dogs (DD). The insertion and use of the device were straightforward and well tolerated by the dogs. Some device-related issues, such as sensor dislocation and trouble with daily calibrations, were reported. A good correlation between the glucose values measured by this CGMS and those obtained with a flash glucose monitoring system and a portable-blood glucose meter, previously validated for use in DD, was found (rs = 0.85 and rs = 0.81, respectively). The functional life of the sensor was 180 days in two of the three dogs, and the use of the device provided high satisfaction to the owners. This innovative device might be considered a future alternative for continuous glucose monitoring in dogs with diabetes mellitus.

**Abstract:**

The novel Eversense XL continuous glucose monitoring system (Senseonics, Inc., Germantown, Maryland) has recently been developed for monitoring diabetes in humans. The sensor is fully implanted and has a functional life of up to 180 days. The present study describes the use of Eversense XL in three diabetic dogs (DD) with good glycemic control managed by motivated owners. The insertion and use of the device were straightforward and well tolerated by the dogs. During the wearing period, some device-related drawbacks, such as sensor dislocation and daily calibrations, were reported. A good correlation between the glucose values measured by the Eversense XL and those obtained with two commercially available devices, previously validated for use in DD, was found (r_s_ = 0.85 and r_s_ = 0.81, respectively). The life of the sensor was 180 days in two of the DD and provided high satisfaction. This innovative device might be considered a future alternative for home glucose monitoring in DD.

## 1. Introduction

**Figure 1:**
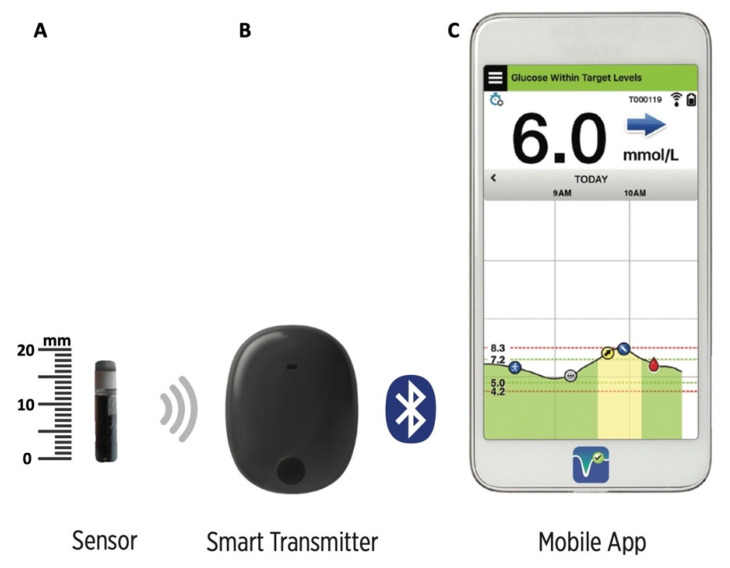
Eversense XL consists of the following: (**A**) the sensor (18.3 mm in length and 3.5 mm in diameter) which is implanted in the subcutaneous tissue; (**B**) the smart transmitter; (**C**) the mobile application which displays glucose information on a handheld device.

Glycemic control is a crucial aspect of the management of diabetes mellitus (DM) and is essential for the prevention of complications in both human and veterinary medicine. Continuous glucose monitoring systems (CGMSs) are frequently used in humans with diabetes, and clinical studies have shown they are effective in reducing hypoglycemia and improving glycemic control [1,2,3,4,5,6,7,8]. Thanks to their high performance, CGMSs have gained popularity among veterinarians and are increasingly being used in diabetic dogs (DD) and cats [9,10,11,12,13,14,15,16,17,18,19]. CGMSs measure, using a transcutaneous sensor, interstitial glucose (IG) concentration, which reflects the blood glucose (BG) concentration [10,20,21]. CGMSs provide sensor glucose levels in real time and allow detection of hyperglycemic and hypoglycemic episodes which might otherwise be undetected [22]. Commercially available sensors have a functional life of up to 14 days and are well tolerated by DD [15]. A novel CGMS equipped with a long-term sensor (Eversense XL; Senseonics, Inc., Germantown, Maryland) has recently been licensed for use in the European Union (CE marking in 2017) [23]. This system consists of a fully implanted sensor, a wearable transmitter, and a mobile application (Figure 1) [23].

The main advantages of Eversense XL are the extended life of up to 180 days, the reduced need for sensor replacement, and the flexibility of being able to remove the external transmitter [23,24,25]. However, unlike the transcutaneous CGMSs, the long-term sensor has to be implanted and removed from the skin by means of a minimally invasive surgical procedure performed by a health care professional [23]. Recent investigations have shown that Eversense is safe and accurate for use in humans with diabetes, the overall mean absolute relative difference (MARD) ranges from 8.5 to 9.4% and the 20/20% agreement rate is 93 and 99% of values in zones A and B on the Clarke Error Grid, respectively [25,26,27,28,29]. To date, no studies have evaluated the use of the long-term sensors in DD. The present case series describes, for the first time, the clinical use of Eversense XL in three DD, and the correlation between IG measured by Eversense XL, IG measured by a flash glucose monitoring system (FGMS; FreeStyle Libre, Abbott, UK) and BG measured by a portable-blood glucose meter (PBGM; Alphatrak2, Zoetis/Abbott, UK) previously validated for use in DD [15,30,31].

## 2. Clinical Cases

### 2.1. Case Descriptions

#### 2.1.1. Dog 1

A 14-year, 2-month-old, 5.3 kg male neutered Yorkshire Terrier was presented to the Veterinary Teaching Hospital of the University of Bologna (UVTH) for a routine re-evaluation of diabetes. The dog had been diagnosed with DM 5 years earlier and, at the time of the presentation, was on a moderate-carbohydrate, moderate-fiber prescription diet (Diabetic Royal Canin, Royal Canin SAS, Milano, Italy) and 4 U of porcine lente insulin (Caninsulin, MSD, Boxmeer, The Netherlands), twice daily. The owner described the absence of symptoms related to DM (e.g., polyuria, polydipsia, polyphagia, weight loss), and was monitoring the BG of the dog by means of blood glucose curves (BGCs) at home, using the PBGM. In addition, the owner often applied the FGMS at least once a month to monitor the IG continuously. On physical examination, the dog had a normal body condition score (BCS, 5/9) and a mature bilateral cataract. The CBC and the biochemistry profile were unremarkable. Urinalysis showed a urinary specific gravity (USG) of 1.030 and glycosuria.

#### 2.1.2. Dog 2

A 12-year, 6-month-old, 23.9 kg male neutered English Setter was presented to the UVTH for a re-evaluation of diabetic control. The dog had been diagnosed with DM 1 year earlier and, at the time of the presentation, was on a moderate-carbohydrate, high-fiber homemade diet and 14 U of porcine lente insulin, twice daily. The DM-related signs were not reported. The owner was monitoring the IG of the dog using the FGMS. On physical examination, the dog had a normal BCS (4/9), and no abnormalities were detected. The CBC was unremarkable and the biochemistry profile showed a marked increase in serum cholesterol (594 mg/dL, reference range [RR] 123–345) and triglycerides (672 mg/dL, RR 30–120). Urinalysis showed a USG of 1.045 and the absence of glycosuria.

#### 2.1.3. Dog 3

A 12-year, 7-month-old, 15.3 kg female spayed mixed breed dog was presented to the UVTH for a routine re-evaluation of DM. The dog had been diagnosed with DM 2 years earlier and, at the time of the presentation, was on a moderate-carbohydrate, moderate-fiber prescription diet (Diabetic Royal Canin) and 6 U of neutral protamine Hagedorn (NPH) insulin (Humulin I, Eli Lilly, Sesto Fiorentino, Italy), twice daily. In addition, the dog was receiving bezafibrate (10 mg/kg; once daily) for the treatment of hyperlipidemia. At the time of presentation, the DM-related symptoms were not reported. The owner was monitoring the IG using the FGMS. On physical examination, the dog had a normal BCS (4/9) and a mild bilateral cataract. The CBC was unremarkable and the biochemistry profile showed a mild increase in alanine aminotransferase (ALT) (106 U/l, RR 15–65) and serum cholesterol (409 mg/dL, RR 123–345). Moreover, a moderate increase of serum triglycerides (417 mg/dL, RR 30–120) was also detected. Urinalysis was not performed.

### 2.2. The Eversense Long-Term Implantable CGMS

The components of the Eversense XL are shown in Figure 1. The sensor is encased in biocompatible material and utilizes a unique fluorescent, glucose-indicating polymer. A light-emitting diode embedded in the sensor excites the polymer, and the polymer then rapidly signals changes in glucose concentration via a change in light output. The measurement is then relayed to the smart transmitter. The sensor has a silicon collar containing dexamethasone, which is slowly released to reduce the inflammation which could degrade sensor functioning. The transmitter is a reusable device worn externally over the inserted sensor which powers the sensor and sends glucose information to the mobile application via bluetooth low-energy technology every five minutes. It is held in place with a mild silicone-based adhesive and is rechargeable via a micro-USB cable. The transmitter and sensor use an inductive link to communicate across the skin (near-field communication). The mobile application needs to be run on a compatible handheld device to receive and display the sensor glucose data from the smart transmitter. The data are stored, for up to a year, on a cloud-based platform and are analyzed by dedicated software (Data Management System, DMS) which is easily accessed by patients and health care providers and generates summary glucose reports (ambulatory glucose profile and other customized reports) [23,24,25]. The detection limits of the sensor are between 40 and 400 mg/dL; when the IG concentration is <40 mg/dL and >400 mg/dL, the mobile application shows “LO” and “HI”, respectively.

### 2.3. Sensor Insertion and Follow-Up Assessments

**Figure 2:**
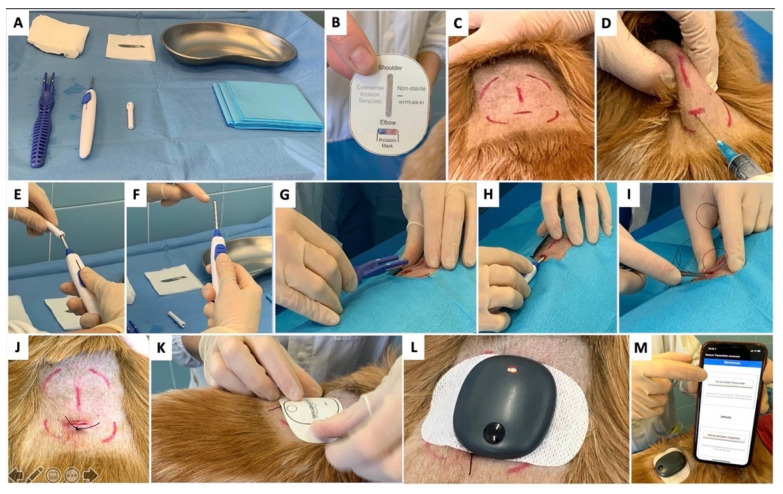
Eversense XL insertion in a diabetic dog. (**A**) Eversense XL sensor pack, blunt dissector and insertion tool with the necessary equipment: sterile cloth, gauze pads (3 with alcoholic chlorhexidine), and scalpel blade; (**B**) the incision template which is used to guide and mark the incision area on the skin surface by aligning the marking template to the marked outer edges of the smart transmitter; (**C**) the skin is trichotomized, marked using the incision template, and cleaned with chlorhexidine (**D**) local anesthesia (lidocaine) is injected along the planned incision site; (**E**) the sensor holder is slid into the insertion tool; (**F**) the sensor is secured inside the insertion tool; (**G**) Once the insertion area is sufficiently anesthetized, a small incision of 5–8 mm is made and, a subcutaneous pocket is created to accommodate the sensor using the blunt dissector. (**H**) the sensor is placed in the subcutaneous pocket making use of the insertion tool; (**I**) the skin is closed with non-absorbable sutures; (**J**) the sensor is now in place and ready for connection with the smart transmitter; (**K**) an adhesive patch, which attaches to the skin and to the back of the smart transmitter, is applied over the insertion site; (**L**) the smart transmitter is placed over the adhesive patch (the transmitter and adhesive patch are removed every 24 to 72 h in order to recharge the battery); (**M**) The smart transmitter is paired with the mobile device and linked to the sensor.

Due to the high motivation of the owners and the good attitude of the three dogs to wearing the FGMS, the subcutaneous insertion of Eversense XL was proposed; written informed consent was obtained. The sensor insertion was performed as described in Figure 2.

**Figure 3:**
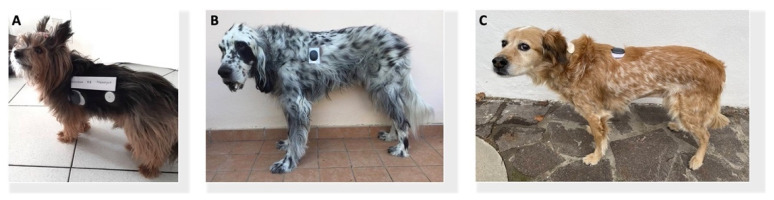
(**A**) Dog 1 wearing Eversense XL (**left**) and FreeStyle Libre (**right**); (**B**) Dog 2 wearing Eversense XL; (**C**) Dog 3 wearing Eversense XL (**right**) and FreeStyle Libre (**left**).

In dogs 1 and 2, the sensor was inserted in the lateral aspect of the thorax (Figure 3A,B). In dog 3, the sensor was implanted in the dorsal aspect of the thorax (Figure 3C).

In dog 1, due to the dog’s poor compliance, the procedure was performed under brief general anesthesia while, in dogs 2 and 3, the procedure was performed under local anesthesia. In the latter case, no signs of discomfort or pain were noted. The device initialization phase consisted of 4 glucose calibration tests carried out 2 to 12 h apart. The device was subsequently calibrated every 12 h by the owner using BG or IG, measured by the PBGM and the FGMS, respectively.

No serious adverse events (AEs) were reported in any of the dogs. In dog 1, a mild erythema in the site of application of the FGMS was noted. In dogs 1 and 3, mild and moderate dislocation of the sensor from the implantation site were noted, respectively. In dog 2, excessive dislocation (i.e., movement of the sensor away from the implantation site) of the sensor affecting the connection with the transmitter was noted. The glucose data were monthly remotely assessed using the cloud-based Eversense DMS and the treatment was changed accordingly. The follow-up visits were scheduled at 90 and 180 days after the sensor implantation. At the time of the last follow-up visit, the owners of dogs 1 and 3 were very satisfied (N 5 on the 5-point satisfaction scale). Dog 1 had no diabetes-related clinical signs and was still receiving 4 U of porcine lente insulin. Dog 3 also did not show clinical signs, and the dose of NPH insulin was 7 U. In dogs 1 and 3, the sensor remained functional throughout the entire expected wear time and, at the end of its lifespan, it was not removed from the subcutaneous tissue. In dog 2, after 20 days, the owner reported trouble with daily calibrations due to sensor dislocation. At the 90-day follow-up visit, the owner was no longer using the Eversense CGM; however, the device was not removed from the subcutaneous tissue.

### 2.4. Glucose Data Analysis

**Figure 4:**
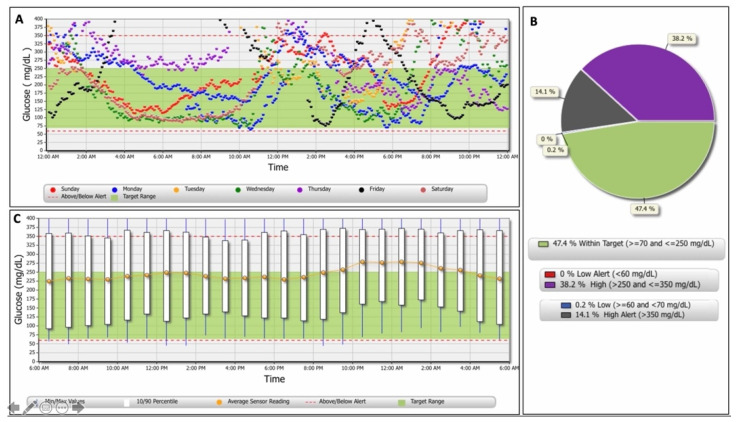
Glucose reports generated by the Eversense Data Management System: (**A**) Glucose trend, divided by days, over a 7-day period. Individual days can be added or removed according to the preferences of the health care providers; (**B**) Glucose pie chart showing the percentage of glucose readings within set ranges over a 180-day period; (**C**) Glucose variability reports over a 180-day period. The legend is represented in the lower part of each report.

Glucose data, automatically calculated by the Eversense DMS, are shown in Table 1 and Figure 4. Eversense DMS data was not available for dog 2, due to the intermittency of recording and short wearing time of the sensor.

Due to the lack of data regarding the accuracy of Eversense XL in DD, the glucose concentrations were measured in all three dogs at least once a month by means of BGCs or using the FGMS. In order to compare the IG detected by Eversense XL with both the IG detected using the FGMS and the BG detected using the PBGM (Alphatrak2), paired glucose values were collected. The BG was measured from the inner pinna with the PBGM, and simultaneously (within 1 min) the IG detected by Eversense XL was recorded from the mobile application. The simultaneous IG levels provided by the mobile applications of Eversense XL and the FGMS was recorded. The owners were allowed to obtain paired measurements during the entire wearing period of Eversense XL. The measurements obtained for the calibration tests and all glucose concentrations above or below the detection limit of the sensor (<40 and >400 mg/dL, respectively) were excluded from the analysis.

The normality of the glucose values was assessed using the Shapiro–Wilk test, and nonparametric tests were used accordingly. The correlation between the glucose concentrations measured by Eversense XL and those measured by the FGMS and the PBGM was evaluated using Spearman’s rank correlation. The differences between the BG measured by the PBGM and the IG measured by Eversense XL were plotted against the reference values in Bland–Altman plots. Analytical accuracy was determined by calculating the mean absolute relative difference (MARD), median absolute relative difference (mARD), mean absolute difference (MAD), and mean relative difference (MRD) [32]. All these are measures of the average difference between Eversense XL and reference results (PBGM). MARD and mARD measure the size but not the direction (higher or lower) of the differences compared with the reference (absolute) as a percentage of the reference value (relative). MAD is similar, but just reports the size of the difference (it is not reported as a percentage), and is commonly used to assess accuracy at low BG values (<100 mg/dL). MRD measures the size and direction of the difference compared with the reference as a percentage of the reference value. Second, analytical accuracy was estimated based on ISO 15197:2013 criteria, which state that at least 95% of results must be within ±15 mg/dL of the BG concentration for BG concentration <100 mg/dL and within ±15% of the BG concentration for BG concentration ≥100 mg/dL. In addition, the precision absolute relative difference (PARD) was calculated. Instead of sensor-to-BG differences, the sensor-to-sensor differences (Eversense XL vs. FGMS) were calculated as the difference between sensor readings divided by the average of the sensors’ readings [33].

Parkes Consensus Error Grid analysis (EGA) for type 1 DM was performed to assess clinical risks for each measurement, and the values of glucose concentrations measured by the reference method (PBGM) were assigned to the x-axis versus glucose concentrations measured by Eversense XL on the y-axis in eight concentric zones with no discontinuities (A through E) defined by different lines [34].

**Figure 5:**
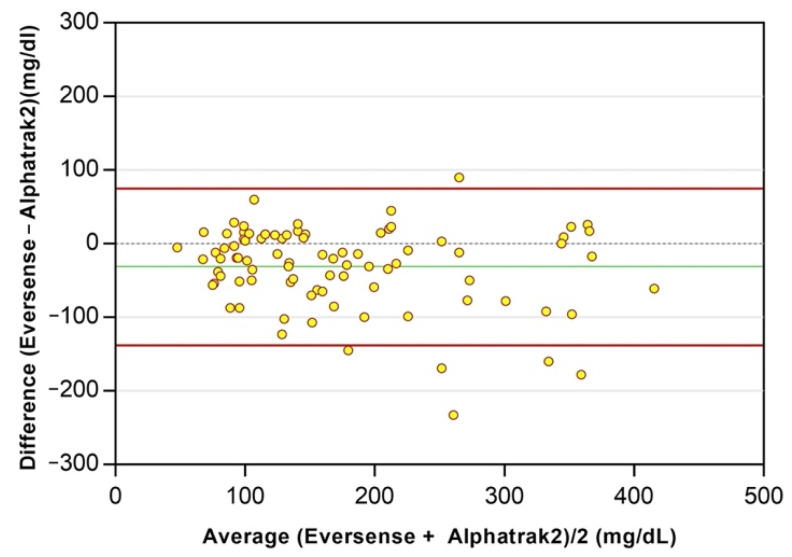
Bland–Altman plots represent the differences between the glucose concentrations obtained using Eversense XL versus those obtained using the PBGM (Alphatrak2) in all dogs. The PBGM glucose values plotted against absolute errors for each corresponding value are on the x-axis. The black dotted line represents a mean difference of 0 between the glucose concentrations being compared. The green line represents the mean difference between the glucose concentrations being compared, and the red lines represent the 95% limits of agreement.

A total of 264 paired glucose results were obtained, of which 66% (175/264) were obtained with the FGMS and 34% (89/264) with the PBGM. A strong positive correlation was found between the IG measured by Eversense XL and the FGMS (r_s_ = 0.85; 95% confidence interval [CI], 0.80–0.89; *p* < 0.0001), as well as between the IG measured by Eversense XL and the BG obtained using the PBGM (r_s_ = 0.81; 95% CI, 0.72–0.87; *p* < 0.0001). The mean ± standard deviation (SD) differences between the BG and the IG were −31.5 ± 54.5 mg/dL (95% limits of agreement, −138.3–75.2; Figure 5). The results of Eversense’s analytical accuracy in the low (BG < 100 mg/dL) and high glucose range (BG ≥ 100 mg/dL) are shown in Table 2. The PARD was 26.3%.

**Figure 6:**
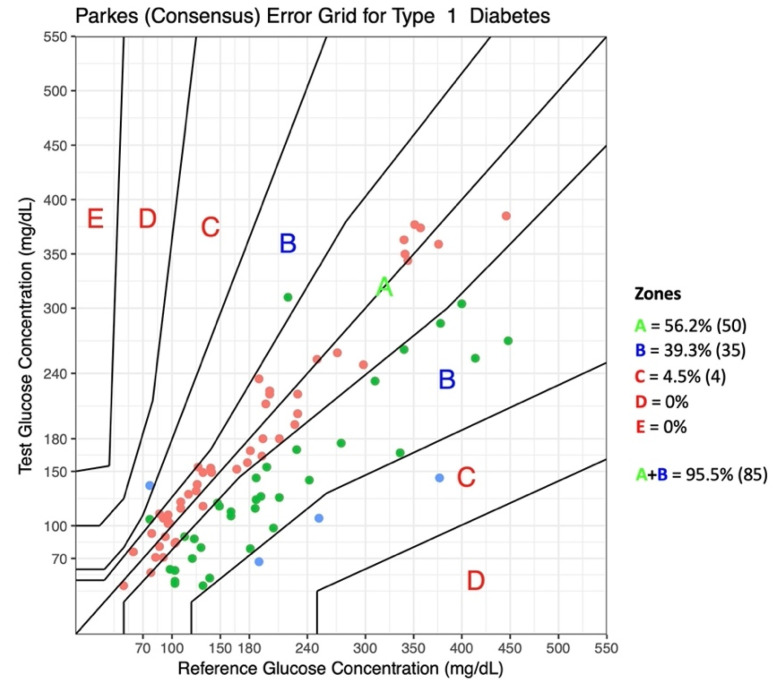
Parkes consensus error grid analysis (EGA) representation with the percentage of values within different zones. The reference glucose values (blood glucose obtained by a portable glucometer) on the x-axis are plotted against the interstitial glucose measurements obtained by the Eversense XL on the y-axis. The different zones designate the magnitude of risk: no effect on clinical action (Zone A); altered clinical action, little or no effect on the clinical outcome (Zone B), altered clinical action, likely to affect the clinical outcome (Zone C); altered clinical action, could have a significant medical risk (Zone D); and altered clinical action, could have dangerous consequences (Zone E). ISO 15197:2013 requires that 99% of the values fall within Zones A + B for a device to be considered accurate.

Evaluation of data using the Parkes consensus EGA showed that 95.5% of the Eversense XL results fell in zones A and B (Figure 6).

## 3. Discussion

The clinical use of Eversense XL resulted in continuous IG monitoring over a 180-day period and high user satisfaction in 2/3 DD. The results showed a good correlation between the glucose concentrations measured by Eversense XL and those obtained using a PBGM and a FGMS, previously validated for use in DD [15,30,31].

Eversense XL is an innovative system for monitoring IG in patients with diabetes [23]. This device overcomes some of the limitations of transcutaneous CGMSs, such as trouble inserting the sensor, insertion pain, the burden of frequent sensor replacement, discomfort from wearing the sensor, dissatisfaction with wearing diabetes devices, sensor dislodgement and skin irritation [24,35,36]. In the present study, the device was well tolerated by all the dogs, and no serious AEs (i.e., discomfort, local and systemic signs of inflammation) were recorded. It is worth mentioning that mild erythema at the site of the application of the FGMS was noted in one of the dogs. Dermatologic complications associated with the use of FreeStyle Libre have been reported in up to 80% and 18% of DD and cats, respectively [15,37,38] Allergic contact dermatitis, likely caused by the sensor’s built-in adhesive, is a known problem in human medicine and, in a retrospective study, it was observed in 3.8% of 1036 diabetic patients using FreeStyle Libre [39]. In a recent study, the incidence of device-related AEs was low in 3023 patients using Eversense XL [29]. In that study, the most frequently reported AEs were sensor location site infection (0.96%), inability to remove the sensor at the first attempt (0.76%) and adhesive patch location site irritation (0.66%) [29].

In the current study, sensor dislocation and trouble with daily calibration were the only device-related AEs reported by the owners. However, since the sensor was not removed at the end of the wearing period, it was not possible to assess whether the inability to remove the sensor could represent a device-related drawback also in DD. The dislocation of the sensor from the implantation site has not been described in humans [29]. Potential reasons for this discrepancy were the different sensor implantation sites (i.e., the thorax in dogs as compared with the upper arm in humans) and the anatomic differences between human and dog skin [23,40]. Of note is that the external surface of the implanted sensor has a silicone collar which slowly releases the dexamethasone acetate into the adjacent subcutaneous tissue to suppress inflammation and the foreign body immune response [41]. This safety mechanism does not allow the formation of a surrounding fibrous capsule which could potentially block the sensor at the implantation site. Sensor movement was less marked in the only small-breed dog included (Dog 1), suggesting individual variability possibly related to dog breed and size. The dislocation of the sensor makes it difficult to carry out daily calibration tests since connection between the sensor and the transmitter is possible only when the transmitter is positioned directly over the sensor. Furthermore, when daily calibration tests are not completed within a 24-h period, the system re-enters the initialization phase [42]. Based on the above, a constant commitment from the owner is required for management of the device.

In the present study, although only dogs accustomed to wearing the FGMS and motivated owners were selected, in one of the three dogs, the Eversense XL was used only for a limited period. This highlights the importance of careful dog selection when this device is used. In addition, the high cost and limited availability of the device are additional factors which should be taken into consideration.

The application of the sensor was minimally invasive, quick and simple owing to the insertion tools provided by the manufacturer. In two dogs, the sensor was inserted under local anesthesia whereas, in one case, due to the dog’s poor compliance, the procedure was performed under general anesthesia. When the procedure was carried out under local anesthesia, no signs of discomfort or pain were noted. In contrast with the FreeStyle Libre, which is often applied on the neck to allow the sensor to be secured with a protective bandage, in the present cases, the thorax was chosen as a positioning site since it allowed for easy implantation of the sensor. Moreover, since the transmitter can be easily removed from the skin, it is not essential to apply a protective bandage.

We found a strong positive correlation between the glucose concentration measured by Eversense XL and that measured by the FGMS and the PBGM. However, the correlation coefficient found in this study was slightly lower compared with those of previous studies validating the FGMS in outpatient DD (r = 0.94 and 0.93) [15,31]. Furthermore, the mean difference between BG and IG was greater than previously reported (Eversense XL, −31.5 ± 54.5 vs. the FGMS, 2.3 ± 46.8 and 17.2 ± 39.0) [15,31]. Possible explanations for this discrepancy are differences in the gold standard methodology for BG measurement (PBGM vs. automated biochemistry analyzer) and in the number of dogs included. Our study is the first to report some preliminary results regarding the clinical and analytical accuracy of the Eversense XL in DD. The device does not fulfill the ISO 2013 accuracy requirements and MARD was higher than that previously reported in humans with diabetes (24.5% vs. 8.5 to 9.4%) [25,26,27,28,29]. Although an accuracy analysis was conducted, the validation of Eversense XL was beyond the scope of this study given the small sample size and the absence of a standardized protocol for measuring paired glucose values. Additional studies, with a larger cohort of dogs, are needed to determine the clinical and analytical accuracy of this device in DD.

## 4. Conclusions

In conclusion, this novel long-term implantable CGMS appeared to be well tolerated and strongly correlated with two commercially available devices previously validated for use in DD. In general, the Eversense XL might be considered a future alternative for home glucose monitoring and could positively impact the adherence and long-term use of CGMSs in DD. However, the use of this device in veterinary medicine could have some limitations, such as excessive movement of the sensor, the need for daily calibrations, high costs, and limited availability. Further investigations are needed to determine the accuracy of the Eversense XL in DD.

## Figures and Tables

**Table 1:** Glucose data in two dogs with diabetes mellitus at the end of the sensor wearing period (180 days).

Glucose (mg/dL)	DOG 1	DOG 3
Mean glucose (±SD ^1^)	119 ± 49.8	249 ± 87.4
Lowest sensor glucose	40	44
Highest sensor glucose	350	399
Total number of glucose values	22.022	9.151
% within glucose target (70–250)	86.1	47.4
% below glucose target (<70)	11.9	0.2
% above glucose target (>250)	2.1	52.3
% below low-glucose alert (<60)	8.8	-
% above high-glucose alert (>350)	-	14.1

^1^ SD, standard deviation.

**Table 2:** Eversense’s analytical accuracy in the low (BG < 100 mg/dL) and high glucose range (BG ≥ 100 mg/dL).

**Low Glucose Range (BG < 100 mg/dL)**	
Number of glucose values	17
MAD (mg/dL)	17.4
Percent of values within ±15 mg/dL of the BG value	52.9% (9/17)
**High Glucose Range (BG > 100 mg/dL)**	
Number of glucose values	72
MARD (%) mARD (%) MRD (%) Percent of values within ±15% of the BG value	24.5
	20.5
	−18.7
	41.7% (30/72)

Abbreviations: BG, blood glucose; MAD, mean absolute difference; MARD, mean absolute relative difference; mARD, median absolute relative difference; MRD, mean relative difference.

## Data Availability

Not applicable.
